# Dosimetric effects of manual cone‐beam CT (CBCT) matching for spinal radiosurgery: Our experience

**DOI:** 10.1120/jacmp.v12i3.3467

**Published:** 2011-04-13

**Authors:** Eduard Schreibmann, Tim Fox, Ian Crocker

**Affiliations:** ^1^ Department of Radiation Oncology Emory University School of Medicine Atlanta GA 30322 USA

**Keywords:** stereotactic radiotherapy, patient positioning, accuracy

## Abstract

Radiosurgical treatment of cranial or extracranial targets demands accurate positioning of the isocenter at the beam and table isocenter, and immobilization of the target during treatment. For spinal radiosurgery, the standard approach involves matching of cone‐beam CT (CBCT) in‐room images with the planning CT (pCT) to determine translation and yaw corrections. The purpose of this study was to assess the accuracy of these techniques compared to advanced automatching using mutual information metrics, with consideration given to volume of interest (VOI) and optimizing translations and rotations in all axes. The dosimetric consequences of our current standard matching techniques were also evaluated. Ten consecutive spinal radiosurgery patients treated in the last year were subjected to analysis. For purposes of this analysis, the automatch using mutual information and a VOI was considered to create “the true isocenter” for positioning the patients. Review of the imaging from this automatch confirmed perfect superimposition of the two datasets within the VOI. Matching the CBCT to the pCT using the automatch allowed assessment of the rotations which had been previously ignored. Recalculation of the dose volume histogram was undertaken for each patient, assuming displacement of the true isocenter to the treated isocenter. Comparisons between the delivered doses and the intended doses were made. The mean absolute lateral/vertical/longitudinal translations and vector displacement between the manual CBCT‐pCT matching isocenter and the true isocenter were 0.13, −0.05, and −0.39 mm, with a minimum and maximum individual pixel vector shift of 3.2 and 8.94 mm. The mean pitch, yaw, and roll correction for automatch was −0.30°, 0.25°, and 0.97° with a maximum of 1.65°, 2.92°, and 1.43°. Four of ten patients had a significant change in the coverage of the tumor due to lack of correction of translational and rotational errors. The largest errors were observed in patients with small and irregular target volumes. Our initial results show that precise positioning for spinal radiosurgery cannot be accomplished with manual pCT‐CBCT matching without a clinical strategy to compensate for rotations. In the absence of this, significant underdosing of the tumor may occur.

PACS number: 87.55.Qr, 87.57.uq, 87.55.km

## I. INTRODUCTION

The aim of extracranial stereotactic radiation surgery (ECSRS) is to deliver high doses of radiation to small tumors adjacent to critical structures. Accurate patient positioning is the key aspect of this treatment modality which requires submillimeter patient positioning.

To ensure proper localization and positioning, a common practice is to check patient position on the treatment table by comparing electronic portal images with digitally reconstructed radiographs (DRRs) generated from the planning CT (PCT) data. If a significant difference is observed, corrections are made by shifting the isocenter until the two images are aligned. However, portal images are projected images and cannot fully visualize soft‐tissue shapes and possible displacement of organs that are volumetric by nature. As organ displacement cannot be fully assessed and corrected by a rigid translation deduced from projected images, a full rigid registration encompassing both translations and rotations and using volumetric images is desirable in order to provide the necessary accuracy in patient positioning for ECSRS treatments.

Recent development of cone beam CT (CBCT)^(^ ^1^ ^,^ ^2^ ^)^ provides unprecedented means for acquiring the volumetric information required for accurate patient positioning to ensure the exquisitely‐shaped ECSRS radiation closely conforms to the tumor while sparing the normal tissue. As an addition to the linac, the CBCT is able to acquire daily volumetric images performed on the patient on the treatment table, obtained just before treatment using doses in the order of a few cGy. The volumetric information provided by the CBCT allows for a complete visualization of the tumor shape and size, allowing for increased treatment accuracy when this information is incorporated into the patient setup process.

**Figure 1:**
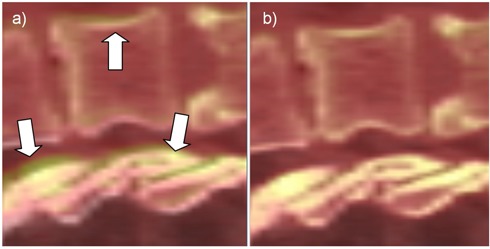
Manual versus ideal registration for Patient #5. Inaccuracies in the manual registrations produced by ignored rotations (a) are marked with arrows. The ideal registration (b) eliminates these discrepancies.

When using CBCT‐based patient repositioning in ECSRS, a 3D volume of the patient anatomy is obtained in the CBCT scan, followed by a volumetric registration that finds the translations and rotations needed to shift the patient to the treatment location as documented in the planning CT dataset. Technically, image registration or alignment is a mathematical procedure that finds coefficients of a transformation matrix, including translations and rotations, which will minimize the anatomical differences between two image sets. Clinically, however, only the translations are considered in the patient setup, as rotations cannot be easily accounted for by the treatment machine's geometry. The translation‐only corrections contain only three parameters describing a common displacement vector between the coordinate systems of the two images and can correct shift only along each axis. Such a registration assumes there are no rotations between the two datasets. An example of ignoring rotations in the patient positioning procedure is illustrated in Fig. 1, where discrepancies at the spinal column level are noted in the translations‐only setup. A patient alignment procedure including both rotations and translations offers a higher degree of accuracy. Such a transform will contain six parameters (three translations+three rotations), and provides a common displacements and rotations vector between the coordinate systems of the two images. Such a complete setup is able to properly correct any rigid changes in patient setup by translating and rotating the treatment table to position the target at its planned position.

Due to the steep dose gradients encountered near the target and critical structures, rotational shifts may have a major clinical effect on ECSRS plan accuracy, but limited data exists on the relevance and magnitude of these rotational errors.^(^ ^3^ ^–^ ^5^ ^)^ Thilmann et al.^(6)^ used bony landmarks in the registration of the CBCT to the pCT. Kriminski et al.^(7)^ reports the Memorial Sloan‐ Kettering Cancer Center experience in using patient positioning based on gold markers matched in MV images with CBCT‐based registrations and replacing it with phantom setups from the same group reported by Chang et al.^(8)^ Guckenberger et al.^(9)^ studied the discrepancies between the EPID‐ and CBCT‐based setup in terms of rotational and translational residual errors. Especially interesting is Guckenberger's report that compares raw data on shift differences for both rotational and translational errors. All these studies give only a raw estimation of shifts and rotational differences, and do not provide an in‐depth analysis of the effect these corrections have on the plan's dosimetric endpoints. Since the effect of rotational errors depends on a structure's position from isocenter – as well as its shape and volume – pure analysis of the raw translational and rotational errors probably does not directly correlate with dosimetric effects on the target and critical structures.

The aim of this study is to dosimetrically quantify the accuracy of the standard CBCT‐based positioning system when only three degrees of freedom are considered. Ten cases of ECSRS treatments are studied, where the standard approach is compared against a full registration considering full rotations and translations. We perform detailed geometrical and dosimetric analysis and on a few cases, where significant underdosing was observed, propose simple methods to ensure proper target coverage.

## II. MATERIALS AND METHODS

Ten spinal radiosurgery patients being treated at our institution were subjected to analysis. To precisely position the patient for the intended radiosurgical treatment, a CBCT was acquired to verify positioning, and treatment of these patients was subsequently delivered according to the manual matching of the CBCT dataset to the simulation CT. The isocenter position and calculated couch translations and rotations obtained in the manual match were recorded in Offline Review (Varian Medical Systems, Palo Alto, CA). The CBCT system is regularly tested for accuracy in accordance with TG‐142^(10)^ using a published protocol.^(11)^ While the accuracy of a SBRT system should be ± 1 mm (as in TG‐142), various parameters were tweaked by Varian engineers on our linear accelerators to yield submillimeter accuracy, which were independently verified by us.

Plans were created using Aria (Varian Medical Systems, Palo Alto, CA). Patient immobilization during treatment was achieved using a SecureVac Vacuum Immobilization Cushion device. A CTV to PTV margin of 2 to 5 mm was used, depending on the complexity of the clinical case. The dose was computed and optimized at a 2 mm resolution grid. All segmentation presented in this study was done on the CT datasets and subsequently transferred to the CBCT datasets using either three or six degrees of freedom registration schemes described below. For increased accuracy, the analysis was performed in patient coordinates with linear interpolation of the dose grid used to obtain dose values at any location.

These same image sets were imported into Velocity AI (Velocity Medical Solutions, Atlanta, GA), which embeds a robust registration algorithm considered to create “the true isocenter and rotations” needed for positioning patients. The registration algorithm implemented in Velocity considers, simultaneously, rotations and translations on all axes, and allows definition of a volume of interest to focus the registration on the anatomy surrounding the isocenter only. Review of the imaging from this automatch confirmed perfect visual superimposition of the two datasets within the VOI. The translation and rotation errors from the “true isocenter with its associated rotations” were then calculated for the manual CBCT match. Recalculation of the dose distribution and the dose volume histogram was undertaken for each patient, assuming displacement of the true isocenter to the treated isocenter for manual CBCT matching. Comparisons between the delivered and intended doses were made.

### A. Registration algorithm

The following terminology will be used for the registration procedures and settings mimicking the clinical and ideal patient setup:

**Clinical** – reproduces the translation‐only alignment used in clinical practice. The CT images have been aligned to the CBCT dataset by an expert (IC) who carefully matched the interface between target and critical organs.

**Ideal** – this is an automated registration searching for both rotations and translations based on a voxel‐based mutual information metric that is optimized by a gradient‐descent algorithm. A volume of interest (VOI) around the PTV is used to factor out irrelevant regions.

In the automated setup, the optimal coefficients of the transform representing the values of rotations and translations for each axis are obtained by an optimization procedure that iteratively adjusts the coefficients of the transformation matrix until the CBCT and CT images match. The match is judged by a mathematical function constructed from the voxel intensities in the two images that describes the desired match. For this particular analysis, we selected the Mattes^(12)^ formulation of the mutual information because it is better suited to deal with differences in Hounsfield units (HU) calibration as compared to single‐modality metrics such as mean squares. Selected settings for the Mattes metric were 25 bins and 10% sampling of the total number of voxels. The transform parameters that minimize the cost function are found by an optimization procedure using a regular step gradient optimizer with a maximum step of 7 and a minimum step of 0.1. The use of a volume of interest is essential for the automated registration, as it allows the algorithm to consider only voxels within the vicinity of the target volume and ignore nonrelevant changes that would not influence the dose, such as weight loss. If an automated registration on the whole body had been used, the automated registration would naturally try to accommodate all changes, and it would have mismatch target position in an attempt to trade off all aspects of the matched volumes.

All registrations were reviewed by the attending physician. Figure 1 presents an example of typical clinical and ideal registration results showing differences and improved accuracy when considering both rotations and translations. In this figure, the CT dataset is displayed as a semitransparent layer superimposed on the CBCT dataset, with a color coding scheme ranging from red to white for both volumes. When using this visualization setup, imperfect registration can be easily detected by the blur surrounding highly‐contrasted regions, such as the spinal bone. The clinical registration (left) correctly positioned the vertebrae, but ignored possible patient rotations. Indeed, the spine is slightly pitched between the two datasets, as indicated by the regions marked with arrows. When turning on the rotations, the automated registration detected rotations of 1.2°, 1.3°, and −1.1° between the two datasets, signaling that a better match can be obtained in this improved setup. Indeed the blurred regions disappeared as the bone is perfectly aligned. The remaining blur is due to the artifacts in the CBCT dataset and are normal here.

### B. Geometric consequences

When analyzing dosages on a specific structure, raw rotation values are not a good predictor of errors because rotational effects depend on the distance to the center of rotation (isocenter) and are thus variant within the dataset. To fully understand and assess geometrical errors for each organ individually, its shape and location within the dataset has to be considered.

**Figure 2:**
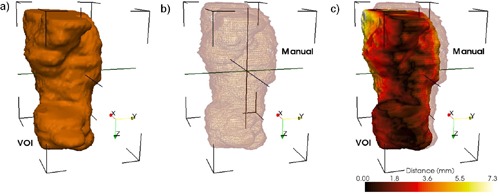
Geometrical differences between the two positioning systems are quantified using a distance tool. The ideal surface is represented as a colored surface, while the surface positioned by the clinical procedure is represented as a wireframe. The color on the surface represents geometrical errors in the clinical patient positioning procedure.

For an accurate analysis we developed a tool that quantifies errors using the structure's segmentation. The tool's functionality and design is illustrated in Fig. 2, where the tumor surface as

positioned by the clinical patient setup is visualized using wireframe. This surface is compared against its positioning when using the ideal setup, show in the figure as a surface rendering. The distance between surfaces positioned by the two matching methods is computed on every point of the structure, and statistics including modal, mean, and maximum are displayed for a quantitative evaluation. Furthermore, structure rendering is color‐coded according to results for error visualization by using brown for minimum discrepancy and white for the maximum. The tool provides a structure‐by structure accurate objective analysis of geometrical discrepancies introduced by the clinical setup, as well as a volumetric visualization of their location.

### C. Dosimetric consequences

The dosimetric accuracy of the clinical patient positioning system is evaluated though dose volume histograms. Organs are positioned relative to the isocenter by the two registration methods, and the contour information is then combined with the original dose matrix to compute relevant dosimetric quantities such as the minimum/maximum dose and DVHs for each organ.

**Figure 3:**
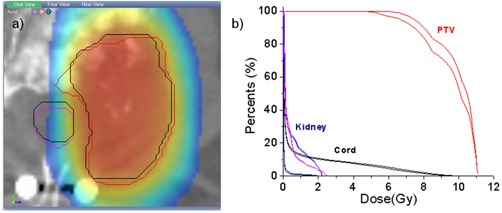
Dosimetric differences between the two positioning systems are quantified using the planning dose matrix: (a) structures positioned by the clinical procedure are shown as colored contours, while their intended location is shown with black contours; the dose is shown as a color wash overlaid. Dose volume histograms (b) can be then constructed for the two datasets to evaluate dosimetric consequences.

This dosimetric evaluation procedure is illustrated in Fig. 3, where a dose wash representation of the planning dose matrix is shown on an axial slice in correspondence with the patient contours aligned by the ideal and clinical methods. The spinal cord and PTV ideal locations are shown in black. For comparison, spinal cord location after the clinical positioning is shown in magenta. In this particular case, a 1.6° pitch and 1.2° yaw rotation is reflected in the displacement of the manually positioned structures from their intended locations. As a consequence, the spine contour is being dragged inside the high‐dose region. Similarly, the PTV (red) was dragged outside the high‐dose region. The dosimetric consequences of these inaccuracies are assessed though DVH plots of the dose coverage on the manually aligned structures. The same dosimetric analysis was repeated for each patient in this study cohort.

## III. RESULTS

Raw differences in translations and rotations between the ideal and clinical positioning methods are shown in Table 1 for the first treatment fraction. The mean differences were 0.12, −0.04, and −0.39, with standard deviations of 1.20, 0.81, and 1.45. The largest discrepancy was of −2.47 on the z‐axis and is attributed to the anisotropic voxel size, with differences on other axes below 1 mm in general.

**Table 1:** Per‐patient differences in translational and rotational shift between clinical and ideal patient setups. Translations are reported in mm, while rotations in degrees.

*Patient #*	*Lat*	*Vert*	*Long*	*Pitch*	*Yaw*	*Roll*
1	1.70	−0.83	0.57	0.81	2.05	1.43
2	0.31	0.38	1.42	−0.44	−1.28	0.55
3	0.74	−0.61	1.94	−1.65	−1.24	0.23
4	−1.36	−0.32	−2.47	−0.04	2.30	−0.60
5	−0.75	−1.00	−0.51	−1.24	−1.35	1.12
6	−1.89	1.66	−0.53	−0.66	0.89	4.14
7	1.31	−0.69	−1.38	−0.37	−2.41	1.40
8	1.40	0.61	−1.50	−0.19	0.73	1.20
9	−0.03	0.28	−1.88	1.11	−0.02	0.05
10	−0.14	0.04	0.43	−0.36	2.92	0.29
Mean	0.13	−0.05	−0.39	−0.30	0.26	0.98
Std. Dev.	1.20	0.81	1.46	0.83	1.81	1.29
Std. Err. Mean	0.38	0.26	0.46	0.26	0.57	0.41

Most translational shifts are correlated with a rotation angle present around one of the angles, indicating the need for a full translation–rotation procedure for an accurate positioning in clinical practice. For example, Patient #4 has the largest translational error but also the largest rotational error. The discrepancies in the translations are thus explained by the presence of rotations, with the result that the physician in the manual match has to try to find a compromise to minimize the effect of rotations by shifting the patient. Almost all patients with rotational errors larger than 2° (Patients #1, #4, #7) had also translational errors introduced by this tradeoff.

**Figure 4:**
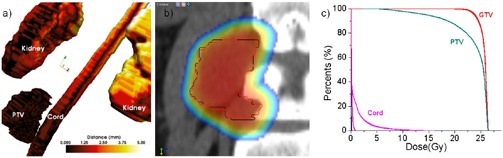
Geometrical and dosimetric analysis for Patient #1: (a) geometrical error with color‐coding quantifying setup error as measured by the distance tool; (b) in a coronal slice, the PTV positioned by the two systems relative to the dose shown as a color wash; (c) DVHs – although the discrepancies were relatively large, the dose was not significantly degraded due to the relatively large and symmetrical target shape.

Large errors do not imply large dosimetric errors. For example, Patient #1 (Fig.4) displayed relatively large rotational errors of 0.80°, 2.05°, and 1.42° on the lateral, vertical, and longitudinal axes, respectively. These errors, however, did not correlate with a significant degradation in target coverage as documented in Fig. 4(c), where the PTV and target DVHs for the clinical and ideal setup are almost indiscernible. This was attributed to the PTV having a large volume (146 cm^3^) and relatively spherical shape. Indeed, the geometrical analysis tools (Fig. 4(a) shows PTV discrepancies of less than 1.25 mm. The rotations and shift are also visible in the dosimetric view (Fig. 4(b), but because of the symmetric and large shape the PTV contour positioned by the clinical procedure (red), the rotations and shift remained inside the coverage area and match the contour positioned by the ideal match (black).

**Figure 5:**
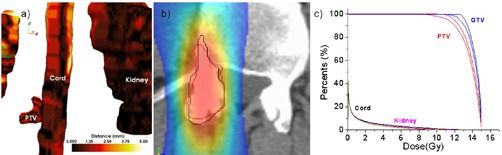
Geometrical and dosimetric analysis for Patient #4. Although the discrepancies between the clinical and ideal setup are mediocre, significant dose degradation was observed due to the target's small size.

Small geometrical errors do not necessarily imply small dosimetric errors. Patient #4 (Fig. 5) has errors of −1.36,0.32, −2.47 mm for translations and −0.04°, 2.29°, and −0.60° for rotations which are not significantly different from the patient previously analyzed. However, the DVH analysis shows significant dose degradation for the target, with coverage decreasing from 94.74% to 90.62% at 12 Gy. We attributed this to the small PTV volume (7.4 cm^3^) having an irregular shape (Fig. 5(a). Indeed, the geometrical analysis documented a maximum geometrical error in the PTV of 3.16 mm. The dosimetric analysis in Fig. 5(b) illustrates that because of the ellipsoidal PTV, any rotation would easily throw the distant ends of the PTV outside the high‐dose region. Indeed, when comparing ideal matching (black line) with the manual matching of the PTV (red line), the latter is less harmonized with the dose distribution's shape and location.

The assumption that the PTV shape and size play an important role is supported also by Patient #3. For this patient, the translational errors were 0.74, −0.61, and 1.94 mm, and the rotational errors were −1.65°, −1.23°, and 0.23°. These are medium range errors, but the PTV itself with a volume of 27.5 cm^3^ is both small and irregular. The dosimetric analysis for this patient is shown in Fig. 3, with the contours positioned by the manual match slightly rotated in the axial plane. This produced a decrease in the DVH coverage from 57.17% to 49.1 % at 10 Gy.

**Figure 6:**
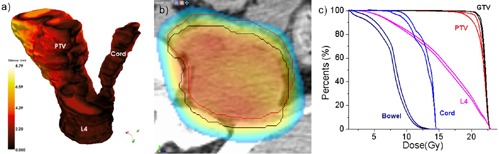
Geometrical and dosimetric analysis for Patient #6. The datasets were aligned at the tumor‐critical structure interface, with a large rotation throwing the upper part of the PTV outside the field.

An interesting case is Patient #6 (Fig. 6), diagnosed with a large tumor of volume 147.1 cm^3^ growing out of the L4 vertebra. His translational errors were −1.89,1.66, and −0.53 mm, displaying also a significant rotation of 4.13° around the z‐axis. Clinically, the CT and CBCT were matched at the level of L4 to minimize the discrepancies at the tumor‐L4 interface using a matching strategy that tried to compromise the rotational error by translations. Because the match focused on the L4‐tumor interface, discrepancies as high as 8.94 mm in the upper part of the tumor far away from the center of rotation were recorded in the geometrical analysis (Fig. 6(a). Dosimetric coverage at the maximum geometrical discrepancy is shown in Fig. 6(b), with the PTV bordering the high‐dose region in the manual match (red). For comparison, its intended position relative to the dose matrix is shown in white. The DVH analysis in Fig. 6(c) showed that the coverage at the prescription dose of 20 Gy to the PTV decreased from 97.27% to 91.86%, while the 95% coverage decreased from 98.28% to 96.60%.

Matching rotational discrepancies for this case is tricky. If we had matched the PTV itself instead of the L4‐PTV interface, this would have created lower dosimetric errors in the PTV, but would have compromised positioning of the L4 vertebra, potentially leading to its overdosage. An alternative would be to match the geometry that is critical, such as the tumor‐L4 interface in this case. Because the tumor is relatively big, we know a priori that the voxels far away from the matching regions will be potentially underdosed. To solve this, we propose using a nonuniform PTV margin that is minimal in those critical regions that we are going to match in the manual registration, and more abundant in the far away regions. This will ensure target coverage if rotations are present. This is a simple and practical solution, as most treatment planning systems allow for definition of nonuniform margins for target volumes, but rely on careful manual matching of the critical region before each treatment.

## IV. DISCUSSION

Radiosurgical treatment of cranial or extracranial targets demands accurate positioning of the isocenter at the beam and table isocenter and immobilization of the target during treatment. Standard localization process for spinal radiosurgery involves initial matching of the kV in‐room images with the planning CT (pCT)‐generated digitally reconstructed radiographs (DRR). This is then followed by manual matching of the CBCT to the pCT.

The accuracy of standard CBCT positioning methods for stereotactic radiotherapy was examined in this work. The first objective was to quantify the residual errors by means of a post‐treatment verification to estimate registration accuracy. For this, we retrospectively analyzed patient positioning based on CBCT in relation to a customized registration, considered as the gold standard. Three‐dimensional image registration of the CBCT image to the planning CT was performed automatically by maximizing the mutual information using a region of interest that included the body of the vertebra containing disease. This registration, used to determine the displacements, was done with three translational degrees of freedom only. A six‐degrees‐of‐freedom registration assessed effects of rotations on geometrical alignment and, consequently, on the dose distribution through DVH plots, with an in‐depth analysis performed on typical cases.

The most important lesson learned from this study is that in the presence of large rotations that are ignored, significant underdosage of the tumor may occur. Overall, even though the difference in planned and delivered dose is small for the majority of patients, over the population we have demonstrated considerable degradation of the delivered dose to the target if the target is large and the match is performed at one of its borders. This is best illustrated on the analysis of Patient #6, where matching the tumor‐L4 interface produced underdosage at the PTV region distant from the center of matching and is thrown out of the treatment field. Based on our data, it may be preferable to customize the margin, with tighter margins near critical structures and a more generous margin further away from them. This is a simple solution, as most treatment planning systems allow for definition of nonuniform margins for target volumes. We feel this is particularly important in order to identify systematic errors prior to the ECSRS treatment, as the plan can be degraded significantly if rotations are not considered in the patient setup. Another strategy to correct for rotations by rotating the treatment couch requires further development of software or hardware solutions to effectively account for table roll and pitch.

Another important observation was that errors depend on tumor shape and size. Not surprisingly, small tumors (for example, Patient #4) were underdosed even if the rotational errors were small. Similarly, we observed underdosage on patients treated with tumors with irregular shape, such as Patient #3. Patients with larger and symmetric tumors did not necessarily display significant dose degradation even for small positioning errors, as show in Patient #1.

The employment of a region of interest and a customized registration were of paramount importance in this work. This is because if deformations, such as weight loss or posture changes, occur between the pCT and the CBCT, an automated registration using the whole dataset will try to accommodate these changes, producing a result that may be suboptimal in the isocenter. If a region of interest is used, the registration metric will focus on the PTV region alone, producing a highly accurate, local registration.

A new treatment couch that is able to correct for both translations and rotations was announced recently,^(13)^ allowing for a seamless integration in clinical practice of the important rotational errors observed in this study. In our study, while the mean pitch, yaw, and roll corrections were −0.3°, 0.25°, and 0.97°, respectively, some of the cases had rotations higher than 1°. This can be easily corrected with the new couch, allowing for a better patient setup when compared to standard treatment couches.

In this work, the original dose matrix was used in the dosimetric analysis. In reality, the delivered dose may vary due to changes in body contour. However, for the patients included in this study we think the changes are not significant. To verify this hypothesis, we selected Patient #6 who displayed both large discrepancies between the ideal and manual registrations, and large variations in the body contour between the CBCT and pCT, as well as having a small PTV volume. The dose was recomputed in our treatment planning using the pCT deformed to the CBCT to preserve HU. The deformable registration used the B‐spline algorithm to model the deformation, the Mattes formulation of the metric function, and the L‐BFGS algorithm to optimize the displacements.^(14)^ No significant changes have been observed between the original and recomputed dose matrixes, with the maximum dose varying from 23.29 to 23.16 Gy and the mean dose from 14.19 to 13.84 Gy. Because these are variations of 0.05% and 2.4%, of the same magnitude with variations introduced by other sources of inaccuracies, we assumed their influence on the dosimetric analysis presented in this work is minimal, and used the original dose distribution throughout the analysis.

To date, the software is used clinically for extracranial treatments, with over 30 patients treated clinically. For each patient, Velocity matches are compared with the OBI system, the two being invariably accurate to within less than 1 mm/1°. Our clinical experience in using Velocity for patient setup was recently reported.^(15)^ The registration module in Velocity was tested using the convergence analysis method, with different registrations started from various initial shifts. For an ideal registration, the same solution should be found by the system, independently of the initial position. For the system used, registration errors in convergence analysis were well below 1 mm/1°.

## V. CONCLUSIONS

It is expected that the CBCT increases the accuracy of patient positioning for ECSRS treatments where precise positioning for spinal radiosurgery cannot be accomplished with manual matching. To achieve similar accuracy as that for cranial radiosurgery, the process mandates a CBCT automatch, using a VOI with correction of rotations. Our initial results of 10 patients show that, in the absence of automated matching methods providing translational and rotational shifts, it is likely that significant underdosing of the tumor may occur. Since a majority of centers do not have a couch with six degrees of freedom, they need to either increase the CTV‐to‐PTV margin in a nonuniform way, or the therapists have to readjust the patient again once that much rotation is observed.
